# Polycystic ovary syndrome: An exploration of unmarried women’s knowledge and attitudes

**DOI:** 10.1016/j.heliyon.2022.e09835

**Published:** 2022-06-30

**Authors:** Eslavath Rajkumar, A. Ardra, G. Prabhu, Vijyendra Pandey, Jeyavel Sundaramoorthy, Rameez Manzoor, K.V. Sooraj, M. Manikandaprabu, Tukaram Badiger

**Affiliations:** a Department of Psychology, Central University of Karnataka, Kalaburagi, India; b Department of Psychology, Central University of Punjab, Bhatindha, India; c Department of Social Work, University of Science and Technology, Meghalaya, India; d Children and Police, State Resource Centre Social Policing, Directorate of Kerala Police, Trivandrum, India; e Department of Psychology, Christ (Deemed to be University) Bannerughatta, Bengaluru, India; f Department of Social Work, Central University of Karnataka, Kalaburagi, India

**Keywords:** Polycystic ovary syndrome, Lifestyle, Awareness, Health and health care behaviours, Regular menstrual cycles

## Abstract

Polycystic Ovary Syndrome (PCOS) is a common endocrine disorder among women of reproductive age and a chief cause of subfertility attributed to ovulation. Besides, lack of knowledge about PCOS, its treatment, and lifestyle changes influence the prognosis. The present qualitative inquiry investigates the knowledge and attitudes of unmarried women towards the syndrome, associated treatment, and necessary lifestyle changes in the fight against the same. A total of 15 participants with PCOS were selected using purposive sampling (n from southern parts of India viz. Kerala and Tamil Nadu states. The telephonic interviews were conducted in late November and early December 2020. He conventional content analysis emerged with six major themes. The themes capsulated women’s knowledge, causes, complications and risk factors, treatment of PCOS their perceived importance of health promotive behaviours such as physical activity, sleep patterns, and perceived support from society. The importance of diet, exercise and a healthy lifestyle were additional relevant factors stressed by the respondents. Although the medicines helped participants attain regular menstrual cycles, they also had side effects reported in the discussion. Few respondents reported that they lacked the necessary awareness of PCOS when diagnosed at a younger age. The study enhances the understanding of PCOS from a qualitative approach that has cultural relevance apart from pertinent clinical and lifestyle implications.

## 1 Introduction

The PCOS, in which ovaries release many immature eggs and eventually turn them into cysts, is prominently influenced by two factors: a) lack of awareness of the syndrome to a great extent and b) changes in one’s lifestyle (Chadha et al., 2019). While awareness of healthy behaviours such as diet has been considered an essential factor in effective monitoring and regulation of the condition, it also plays a crucial role in regulating ‘sex steroid metabolism’ (Sessa et al., 2013). Past studies have shown that a ‘high-lipid and low-fibre’ diet can increase the androgen circulating levels (Pasquali and Casimirri, 1993; Enkhmaa et al., 2018). However, very high lipid intake may decrease Sex Hormone Binding Globulin (SHBG) levels and increase free androgen index. As a result of these complicated clinical manifestations, several conditions are bound to be involved in PCOS. Such conditions mostly are irregularity in menstruation, hyperandrogenism, a complication in pregnancy, infertility, and increased prevalence of obesity, i.e., abdominal obesity in particular (Hart et al., 2004). An increase in adiposity, mainly abdominal, is linked with hyperandrogenemia and increased metabolic risk.

Apart from obesity and ovarian dysfunction, hypothalamic-pituitary abnormalities were also found as risk factors for PCOS (Pitchai et al., 2016). At the same time, in terms of its consequences, past studies showed a tendency for higher rates of miscarriages, fetal deformities, and other complications during pregnancy, including neonatal complications and premature deliveries (Van der Spuy and Dyer, 2004). Yousaf et al. (2013) reported that individuals, despite being aware, tend to avoid medical treatment, wherein they mainly neglect the complications associated with the syndrome. Some reasons for avoidance of medical care were reported in the past. Such reasons included a) unfavourable evaluation of medical care, primarily resultant of concerns over side effects, physicians and healthcare-related factors; b) lesser perceived need, most patients anticipate recovery over the period was another noted reason. Besides, c) traditional barriers such as time constraints and higher medication costs are other reasons (Taber et al., 2014).

In addition, PCOS is also reported to impact the psychological well-being of the individuals and encourages psychological morbidities such as poor body image, self-esteem issues, depression and reduced health-related quality of life in affected women (Barnard et al., 2007). Due to subclinical atherosclerosis, hypertension, hyperlipidemia, endothelial dysfunction, and inflammation, women with PCOS also tend to be at greater risk of cardiovascular disorders (Thomson et al., 2011). However, the illness thus has both short term and long-term consequences that may affect the women unfavourably in various ways and at different stages of their lives. Therefore, it is essential to find effective therapies to prevent, maintain and treat PCOS effectively to decrease the consequences. Also, the diagnosis is vital at the earliest to avoid long-term complications associated with this syndrome.

Experts and doctors should prefer first-aid treatments such as a healthy diet and regular physical exercise for PCOS (Cussons et al., 2005). Though the function of weight loss in improving PCOS status is controversial, weight loss may be helpful in obese or overweight individuals with other health-related issues. Notably, exercise therapy can be very effective in PCOS in managing overweight and obesity. Maiya et al. (2008) argued that physical activities and exercises that do not have any possible side effects should be practised as the first-line treatment for PCOS. Even a minimal amount of weight loss over some time, as little as four weeks, is sufficient to improve the condition of PCOS.

Although PCOS is a common health condition across developed and developing countries with a prevalence ranging from 4% to 12% among the reproductive woman population (Azziz et al., 2004), concerning the Indian context; however, limited studies, mainly using qualitative methods, have been carried out. Nevertheless, although few essential observations made by endocrinologists and gynaecologists show a significant increase in the PCOS cases among women, especially unmarried, it also observed that prospect high-risk individuals are less aware of the syndrome and associated consequences (Nidhi et al., 2011). Provided that PCOS is a common cause of irregular periods among young unmarried women (Choudhary et al., 2017), it indicates the importance of not only disseminating awareness regarding the high risk but also effective management of this fast-spreading contemporary health issue.

Even though a considerable number of studies (Gupta et al., 2017; Choudhary et al., 2017; Dasgupta et al., 2012; Thathapudi et al., 2014) in the Indian context were reported in the literature, those studies, however, were limited to investigating the prevalence, incidents, clinical and biochemical characteristics among adolescent and married reproductive age groups. While a qualitative inquiry into unmarried reproduce age groups has been ignored in the literature, especially in the multicultural Indian context. Although a few studies, such as (Gupta et al., 2015; Shinde and Patil, 2019), have compared unmarried and married women populations with PCOS, such comparisons were limited to exploring differences in clinical characteristics, incident rates and risk assessments. Notably, the women with PCOS for both the aforementioned studies were primarily unmarried than married.

Besides, it was reported in the past that unmarried women with PCOS were at higher risk of being obese and overweight than married women with PCOS (Shaheen et al., 2015). Furthermore, women with PCOS in their reproductive ages are generally more likely to be anxious and depressive (Månsson et al., 2008; Zehravi et al., 2021) due to the complexities of hormonal imbalances, reproductive health, and anticipation of marriage and fertility soon. Thus, it is essential to investigate unmarried women’s awareness of PCOS and explore their perceptions and attitudes toward the associated treatment and necessary lifestyle changes. Qualitative inquiry offers relevant insights into this investigation. Adapting to a qualitative approach such as a semi-structured telephonic interview provides rich data emerging from the non-restricted mode of responses to the intended topic under study.

Consequently, the dire need to explore and adapt to a greatly ignored approach led to this current investigation of PCOS diagnosed individuals' perception of the syndrome, the effective treatment, possible lifestyle modifications, and knowledge of associated aspects is crucial in efficiently managing their conditions. Thus, the current study aims to explore how the perception and knowledge of PCOS, associated treatment, and routine life modifications could bring changes in lifestyles of individuals diagnosed with PCOS. Also, due to limited studies in the area, especially among unmarried women, using qualitative inquiry, this study is of greater relevance in exploring significant insights for the study objectives. Thus, the current study’s findings would considerably be of greater relevance for unmarried females with PCOS in assisting them effectively in dealing with and fighting the syndrome.

## 2 Methods

### 2.1 Research design

A qualitative explorative research design was used in this study to explore the intended objectives by deploying semi-structured interviews via telephone. The semi-structured questions investigated the participants’ perception and knowledge about PCOS and the associated treatment. Also, their respective influence over necessary lifestyle changes for effective management of the syndrome. The interview schedule was developed with the support of an extensive literature review (Barnard et al., 2007; Boomsma et al., 2006; Colwell et al., 2010). Finally, questions were formulated and verified by two experts gynecologist and a researcher working in reproductive health. According to the suggestions and feedback, necessary modifications have been made, and the face validity of the interview schedule has been established. After finalizing the interview schedule, it has been validated by conducting the interviews with a couple of participants.

### 2.2 Participants

Fifteen young female adults (N = 15) diagnosed with PCOS, drawn through purposive sampling from the southern part of India (Table 1) aged between 19 to 25 years, were chosen for the research. The study participants revealed the age of PCOS diagnosis as between 15 – 18 years (N = 8) and 19 years and above (N = 7). With qualifications of under-graduation (N = 9) and post-graduation (N = 6), majority of them reported being students, while two were of the working profession (Students = 13, Working professionals = 2).

**Table 1:** Demographic details of the participants.

Demographics	Variables	Frequency (N = 15)
Education	Under Graduation	9
	Post-Graduation	6
Occupation	Student	13
	Working	2
Residence	Urban	5
	Rural	10
PCOS diagnosed at age	15–18 years	8
	19 and above	7

### 2.3 Recruitment of sample

For the recruitment of a sample for the current study, the researchers approached the participants through their acquaintances and sought details of potential respondents, i.e., the female students and professionals diagnosed with PCOS. Consequently, a pool of fifty likely respondents with contact details was prepared and accordingly was approached individually to invite their participation in the study. However, amidst the pandemic-driven difficulties, only fifteen unmarried women among the fifty potential respondents approached, agreed to participate in the intended interview.

Primarily, unmarried females above 18 years old have been already diagnosed with the PCOS, and are currently undergoing treatment for the same were included in the current study. The disease condition was confirmed by asking self-reported questions like having been diagnosed by a doctor for PCOS, whether they are on medication or not, if yes, those individuals were recruited into the current study.

In comparison, potential respondents from the pool who initially agreed but discontinued the interview for unstated reasons were excluded from the study. The second author conducted the interviews in both Malayalam and English language in accordance with the respondents' convenience. The Malayalam interview transcript was translated into English for uniformity and analyses purpose by an expert who is good at Malayalam and English languages.

### 2.4 Study setting

The intended study was carried out predominantly in the southern states of India – Kerala and Tamil Nadu states. The majority of respondents were from Kerala state, specifically from the six districts: a) Wayanad, b) Kozhikode, c) Palakkad, d) Kannur, e) Kasargod, and f) Patthinamittha. The two working professionals represented Chennai – Tamil Nadu state. Notably, the study setting represented the south Indian cultural setup.

### 2.5 Procedure

By adapting to a purposive sampling method, potential study participants were approached to seek their willingness to participate in the intended semi-structured telephonic interview. The telephonic interview method was chosen due to pandemic-driven restrictions and lockdowns put in place. Prior to the conduction of the telephonic interviews, their convenient time to participate in the study was confirmed. Besides, rapport and trust were built between the respondents and the author who conducted the interviews. They were briefed on the purpose of the study, and their consent was taken to record the telephonic interview and confidentiality about their identity was assured. The respondents were requested to seek clarity for any ambiguous questions being asked to them. Each telephonic interview lasted between 45 and 50 min. The telephonic interviews were conducted in late November and early December 2020. After completing the telephonic interviews, the second author transcribed the interview recordings which were cross-verified by other authors. In addition, after the first three authors analyzed the data, it was reviewed by the other authors. The Departmental Ethics Committee has approved the current study (Ethical Clearance number: CUK/PSY/DERB-2020-21/16 dated 13/11/2020) A few example questions s of the interview schedule have been presented in Table 2.

**Table 2:** Example interview questions.

Sl. No.	Questions
1.	What do you understand by the PCOS?
2.	What do you think are the reasons for developing PCOS?
3.	What treatments are available for PCOS, and what are their processes?
4.	Do you take medications for this condition? If Yes, for how long? Any changes in the condition? If No, what were the reasons for avoiding medications?
5.	How do you think that PCOS affects people?
6.	How important do you think medication and treatment are for PCOS?
7.	How do you manage your condition of PCOS?
8.	How is your family supporting in managing the syndrome?
9.	What lifestyle have you followed before diagnosis? How do you see your lifestyle post-diagnosis of PCOS, and are any changes to report?
10.	Have you felt any Did you feel any change in your attention, concentration, thinking, and decision making due to PCOD?

### 2.6 Data analysis

The conventional content analysis method (Hsieh and Shannon, 2005) was deployed in the study. This qualitative research tool helps establish the meaning through relevant words, themes, or concepts within the given qualitative data. The transcription, classification, and interpretation of the codes were made manually. The recorded interviews were transcribed and processed several times to fully understand the acquired data and ensure the familiarity of the information shared by the respondents. The sentences and paragraphs of the interviews and units of analysis were condensed based on their content and context. The packed meaning units were summarized and labelled with codes using the guidelines offered by Elo and Kyngas (2008). Then, the codes were classified, and subcategories were formed by comparing their similarities and differences. The more frequently used codes that were close to exploring the current study objectives were given more weightage. Subsequently, different codes obtained from the study data were placed into potential themes, sub-themes, and main themes. The multiple authors reviewed the identified themes and sub-themes to ensure that they represented the data. Finally, the themes that expressed the latent content of the text were named and defined in a manner that tapped the essence of the study objectives and research questions directly.

## 3 Results and discussion

The transcribed interviews of the current study yielded the themes and sub-themes. Specifically, the present study's findings (see Table 3) have been reflected in six major themes: a) *Women’s knowledge of the syndrome and perceived need for lifestyle changes,* b) *Women’s personal views towards the syndrome*, c) *Changes experienced due to PCOS*, d) *Perceived side effects of PCOS medications*, e) *Perceived* r*ole of relevant social factors for effective management of PCOS*, and f) *Past experiences of PCOS medications*. Furthermore, relevant sub-themes emerged under these six themes.

**Table 3:** Themes and Sub-themes derived from the study findings.

Themes	Sub-themes
Women’s knowledge of the syndrome and perceived need for lifestyles changes	Knowledge about PCOS
	Knowledge about causes and risk factors
	Knowledge about various PCOS treatments
	Knowledge about health-promoting behaviours
	Lifestyle changes for effective management of PCOS
Women’s personal views towards the syndrome	Perception towards PCOS
	Perception towards PCOS treatment
Changes experienced due to PCOS	Physical changes caused by PCOS
	Cognitive changes caused by PCOS
Perceived side effects of PCOS medications	
Perceived role of social factors for effective management of PCOS	Role of society’s attitudes
	Role of perceived social support
Past experiences of PCOS medications	

Overall, the findings highlighted the perception of individuals diagnosed with various aspects of PCOS. The participant's knowledge of the syndrome, causes, complications and risk factors, treatment, and management of PCOS were major explorations of the findings. Likewise, their perception of health promotive behaviours such as physical activity, sleep patterns, interpersonal relationships, and perceived social support were additional insights of the present study. Initially, the participants appeared to perceive the importance of diet, exercise and a healthy lifestyle in maintaining PCOS and overall health. A lack of awareness in those diagnosed at a younger age became apparent as the study progressed. The comprehensive understanding of PCOS was also noted to be inadequate in society. Though pharmacotherapy helped individuals get a regular menstrual cycle, it also had some adverse effects. Also, participants reported certain unhealthy behaviours or lifestyles which pre-existed in the diagnosis.

Specifically, theme one – *Women’s knowledge of the syndrome and perceived need for lifestyle changes* – comprised of five sub-themes. The five sub-themes include: a) *Knowledge about PCOS, b) Knowledge about causes and risk factors, c) Knowledge about various PCOS treatments, d) Knowledge about health-promoting behaviours, and e) Lifestyle changes for effective management of PCOS.* The second theme - *Women’s personal views towards the syndrome* – included two sub-themes. The two sub-themes were namely: a*) Perception towards PCOS, and b) Perception towards PCOS treatment.*

The third theme - *Changes experienced due to PCOS* – was emerged with respect to two sub-themes namely: *a) Physical changes caused by the PCOS, and b) Cognitive changes caused by the PCOS.* The fourth theme - *Perceived side effects of PCOS medications* – emphasized the perceived side effects of the PCOS medications, especially from the perspective of unmarried women. The fifth theme - *Perceived role of social factors for effective management of PCOS* – emerged with respect to two sub-themes namely: *a) Role of society’s attitudes, and b) Role of perceived social* support*.* However, the essence of these several sub-themes was combined and discussed under respective primary themes.

### 3.1 Theme one: women’s knowledge of the syndrome and perceived need for lifestyle changes

This theme, through the corresponding six sub-themes has highlighted participants’ knowledge about PCOS, the causes, related risks factors, treatment, the health-promoting behaviours, and perceived need for lifestyle changes for effective management of PCOS.

#### 3.1.1 Sub-theme one: knowledge about PCOS

Most respondents described PCOS to be resulting in irregularity in the menstrual cycle, cysts, hormonal imbalance in the body and associated weight gain resulting in changes in physical appearance. However, few participants diagnosed at a younger age were least bothered about the health issue, which could be attributed to the lack of pertinent awareness.

*“PCOS means polycystic ovarian syndrome. It is a common, disorder, like having a cyst in the ovary. Hormonal changes maybe there. It’s because of the hormonal changes and our daily…food and all.”* (Respondent BP).

*“PCOD........Polycystic ovary disease or syndrome. We have cyst in our ovaries which can lead to several problems like irregular menstrual flow, weight gain, etc... Moreover, I do have this syndrome, honestly I don’t know much about it okay..”* (Respondent AS).

*“Actually, as I told you, it was around the age of 15 and at that time, I didn’t think about it much; I just ignored this thing. After some years I got to know it will cost things in future. That’s when I started keeping the diet in control and all those things.”* (Respondent AJ).

These explorations of participants' knowledge about the syndrome were more in line with the key features of the definition offered by Hart et al. (2004). While the respondents' lack of awareness has supported the observation of Pitchai et al. (2016) that about 30% of individuals (in the same Indian culture) were ‘minimally’ and ‘not at all’ aware of PCOS. Further, these findings have also provided empirical support for the past observations of Nidhi et al. (2011) that prospect high-risk individuals are less aware of the syndrome and accompanying consequences. However, unlike Nidhi et al. (2011) whose population was restricted to adolescents, the current study findings offer important insights into the prevalence and knowledge of unmarried reproductive-age women in south India.

Besides, although the present findings validate the conclusions of Pitchai et al. (2016) with perceived awareness of PCOS among reproductive age groups, the current findings offer additional evidence for the same from varied cultural settings, i.e., Southern Indian, especially through a qualitative approach. Moreover, Thus, the current findings warrant adequate attention, knowledge of the syndrome and the importance of education, especially the unmarried women population. It is essential as they are likely to be at higher risk of psychological disturbances (Månsson et al., 2008; Zehravi et al., 2021), especially regarding their reproductive health, i.e., fertility post-marriage shortly.

#### 3.1.2 Sub-theme two: knowledge about causes and risk factors

Above and beyond, the perceived causes of PCOS by the participants were related to unhealthy dietary habits (e.g., consuming junk foods, soft drinks, more non-veg items, fried and oily items, bakery items, not drinking enough water, not consuming more food items with fibres etc.), lack of exercise and physical activities, erratic sleep and stress. Whereas few other study participants, at their younger age, reported having been unaware of the PCOS syndrome and associated complications when they confronted the lab technicians who enquired if they were aware of the same during another testing in the lab such as ultrasound. The respondents shared that they gradually understood the possible risk factors of the syndrome and took necessary measures by themselves. Further, the participants’ knowledge about the related risk of the syndrome seemed to increase with their age and exposure. Particularly with age, they shared that they could gradually understand the severity of the syndrome with improved knowledge and act accordingly. Their maturity also resulted from realising the long-lasting effects of the syndrome.

*“I have many friends who also affected by PCOD. The major reason for PCOD is our food habits, Lifestyle and irregular sleeping pattern.”* (Respondent SR).

*“Haaa... eating more sweets… using more oil and fatty substances in food, fried items. Stress may also add up. Lifestyle changes are the main reason, when I changed my place for studies…. The food and the climate there bring changes in PCOD.”* (Respondent BP).

*“It was noted when I was at the ultrasound centre to check the status of my kidney stones, when the technician asked me whether I have PCOS, but being the 16 year old with no one with PCOS mentioning in the family, I was clueless… Now I am being extra cautious.. with improved knowledge as well as maturity instilled through growing age, I reckon..”* (Respondent PMP).

Pertaining to the prevalence of PCOS among the reproductive age groups, especially among the unmarried women, although past studies (Choudhary et al., 2017) have reported about 24% prevalence rate, limited studies, specifically in the Indian culture, have emphasized the awareness of causes and risk factors among the reproductive age group. It is in this context, the present explorative study from a qualitative perspective goes beyond the observations such as of Choudhary et al. (2017), to highlight the knowledge of reproductive age groups’ unmarried women population in south Indian states. Through reporting the knowledge of PCOS among women, the present study has emphasized that unhealthy dietary habits and lifestyle practices count as major causes and risk factors of PCOS. Thus, it is warranted that the unmarried women population in their early reproductive age need to be educated about the causes and related risk factors that could be prevented in their structured efforts to combat the syndrome.

#### 3.1.3 Sub-theme three: knowledge about various PCOS treatments

On the treatment note, participants reported to have been aware of varied options and approaches such as *Ayurveda*, Homeopathy, Allopathy, and *Unani*. In particular, participants shared their awareness of allopathy medicines to have more side effects than other medicines reportedly. On the contrary, few other respondents shared their knowledge of allopathic medicines' usefulness and minimal side effects. While a few others shared that as per their understanding, allopathic medicines could lead to several health issues such as increased headache, weight gain, mood fluctuations and aggression, ulcers, etc. Whereas for other approaches, such as *Ayurvedic* and *Unani*, participants reported their understanding of the possible discomforts with the same such as the taste of these medicines and strictness in the diet. Meanwhile, for the homeopathic practice, they shared that they were aware of the tendency to be frequently forgetful of the medications.

*“I don’t remember being told/informed that there could much problem in taking the allopathic medicine, because there is only one tablet to take at night and that people with PCOS also need not much be bothered….. While taking the homeo medicines, it was told to be really difficult. There were minimum three different medicines which were mentioned to be taken thrice a day, that many times people would forget to have medicine..”* (Respondent AL).

*“While taking allopathic medicines, I have been told that women are more prone to headache, gaining so much weight and developing ulcer.”*(Respondent BP).

*“No...I haven’t heard or known from any sources that there would be much difficulties taking Allopathic medicines.. But I am aware that the taste for Ayurveda medicines was the major problem with it….”* (Respondent AS).

*“Uhh... It is known to me that the Unani medicines itself were very bad.. that they are very bitter so it could be very hard to consume them.”* (Respondent KM).

Although the past studies such as those of Pitchai et al. (2016) in the Indian context found that about 93% of women in their reproductive age group were aware of various treatment options for PCOS, such exploration was resultant of a quantitative approach and restricted to only one study are, e.g., Mumbai, Maharashtra (Pitchai et al., 2016). It is in this background, with the current study’s qualitative approach and multiple study areas in the southern part of India put in place, the findings offer vital insights into the knowledge of various treatment options, especially from the perspective of unmarried women of reproductive age who are more likely to face fertility complications post-marriage shortly. Therefore, the present findings emphasize educating the pros and cons of various treatment methods viz. allopathy, homoeopathy, Ayurvedic, Unani etc. It is emphasized that such efforts, especially for unmarried women who are in their early reproductive age of early adulthood, should adopt multidisciplinary approaches.

#### 3.1.4 Sub-theme four: knowledge about health-promoting behaviours

The participants reported being aware that dietary habits are one of the leading causes of PCOS. Although diet is a meaningful health-promoting behavioural change to cope with the syndrome, due to its associated difficulties in continuing the same regularly, most participants have reported that they would be more likely to quit the desired diet due to inconsistencies such practices. Participants also shared that, according to their knowledge, eating a lot, especially excessive consumption of oil, fried items, sweets, bakery items, non-veg etc., were known to be worsening the condition of the syndrome. Consequently, although many opined that, as per their understanding, most women with PCOS could not solely avoid the same from dietary habits, a few shared that as per anticipation, some women with PCOS could considerably reduce such consumption, especially when they are in their native place, (unlike far-away for higher studies).

*“It has become very predictable these days, but this was difficult before diagnosis earlier. The PMS issues were reported to be persistence, in my knowledge… along with mood swings and pain and all. But while doing exercises and all, in my understanding, the pain would literally be less.”* (Respondent APM).

*“Haaa... eating more sweets… using more oil and fatty substances in food, fried items. Stress may also increase the severity of the syndrome... Certain changes are the main reason too, For instance, when one changes the place for studies…. The food and the climate there bring changes in PCOD..”* (Respondent BP).

Furthermore, practising self-care and health-protective behaviours are highlighted as the respective areas to be looked upon for effective management of PCOS. Especially when inculcating healthier eating habits in their lives, many participants were of an understanding that they could witness the changes in the severity of PCOS and overall health condition.

In line with previous studies, as shared by the respondents, their knowledge regarding health-promoting behaviours in effective management of PCOS has been a vital finding of the current study as several participants reported their knowledge-driven tendencies to engage in healthy behaviours such as diet and exercise. As per the knowledge of the study respondents, as shared, it indicates that transformation to a health-promoting behaviour can help conquer PCOS. However, they were also aware that individuals with PCOS, including themselves, could find it hard to keep it going for a more extended period.

The current findings, although reports that the respondents have adequate knowledge of the health-promoting behaviours, due to the anticipated inconsistencies in the practice of the same, the findings partially validate the reports of recent research such as Guo et al. (2022) that women in their early adulthood - reproductive age could have poor health-promoting behaviour. Hence, in the background of recent research, the current study could bring up the necessary bridge in enabling the individuals to practice what they know. Moreover, although the result of Guo et al. (2022) could resemble the Indian culture (due to the similarity of the collectivistic culture of China), further studies are warranted to explore the differences within the multicultural context of India.

#### 3.1.5 Sub-theme five: lifestyle changes for effective management of PCOS

Furthermore, in the current study, the PCOS patients have shared their knowledge of the need for lifestyle changes to manage their PCOS condition better. This sub-theme mainly emphasized the importance of engagement in physical activities, adapting healthy sleep patterns, and stress management. Especially, a few respondents have shared their understanding that a lack of physical activities or stopping the regular physical activity suddenly could be the prime reason for the escalation of the severity of the syndrome. Before and after the diagnosis, only a few participants' changes in the sleep cycle were made, but having a regular sleep cycle had been thought to be consistently beneficial among PCOS patients.

*“In my circle, I have known people who were dancing from a very young age till their 10th class, and they seem to have suddenly stopped their dancing practice.. In such cases, I have been told that doing so/engaging such practices can be one main reason for severity of PCOS, because women with the syndrome do not seem to have any other form of exercise....”* (Respondent AS).

*“The cyst was very tiny and if we are doing exercises and all in a regular basis, it won’t happen at all. I think doing exercise is quite good for small cysts. For big one, we may have to take medicines for long.”* (Respondent APM).

*“It is known through my friends who have had PCOS, that in this condition, they were prone to irregular sleeping pattern.. subjected to stressful events....financial issues, esteem issues, parental bickering... difficulties in engaging in any exact physical activity…”* (Respondent PMP).

Predominantly, the relevance of lifestyle changes has been well emphasized by Barnard et al. (2007), who argued that lifestyle changes had improved the metabolic and reproductive manifestations of PCOS (polycystic ovarian syndrome) and improved mood and self-esteem, depression, anxiety, and psychological well-being. Heeding to this observation, the present study findings argue that the lifestyles of PCOS patients, in general, are crucial in determining their condition and are observed to comprise various aspects. According to some respondents’ knowledge of exercising practices around the menstrual cycle, regularity of date could be observed and exercising could be beneficial.

This understanding of the study participants validates the past findings that exercises stimulate oxidative metabolism in tissues and that oxidative metabolism in the ovary can facilitate the development of follicles (Maiya et al., 2008). Hence, the present study, apart from emphasising the knowledge of unmarried women about required lifestyle changes for PCOS management, it recommends explicitly that exercise should be in the structured first-line treatment for PCOS (Cussons et al., 2005). Significantly, these lifestyle changes, potentially flexible and feasible (Arentz et al., 2021), could help individuals obtain desirable expectations such as controlling one’s weight gain and avoiding the tendency to be bound with the condition of obesity eventually. Further, as suggested by Wahrenberg et al. (1999), the study argues that a minimal amount of weight loss over some time, as little as four weeks, is sufficient to improve PCOS condition.

### 3.2 Theme two: women’s personal views towards the syndrome

Personal views of study participants encompassed their perception of the syndrome and its treatment. Most of the respondents had similar perceptions towards the causes of PCOS and its effects on the body etc. Most of them perceived medicines and treatment to be necessary at some stage.

#### 3.2.1 Sub-theme one: perception towards PCOS

In contrast to the usual anxiety, respondents shared their perceptions of mixed feelings post-diagnosis, highlighting the surprise and later joining their peers of similar states to share the common burden and cope with the syndrome. Some participants perceived that the syndrome is only relevant to females in modern era while associating it with the causes such as hormonal and lifestyle changes and considering these experiences as bad in their personal views. The study respondents have regarded the syndrome as a bane to the women, especially concerned to the unmarried women population in their reproductive age. Hence, the respondents have indicated they seem to feel bad and tend to curse themselves for having been diagnosed with PCOS, and they anticipated a psychological need to manage their syndrome.

*“I think it’s very common these days.. For instance, when I got to know about mine.. I felt.. that I am also one among them(friends). This is because everybody in my circle is having PCOS. They would always talk about this, and I would say that I don’t have this; after diagnosis, I called them, and they said, ‘welcome to PCOS family’.”* (Respondent APM).

*“I think it is the female disease or syndrome which we can see only in this modern situation or era. So I think the cause of many hormonal changes as well as our life style and it will affect the people very badly in my perception, because the periods, the first thing or symptoms shown our body is the irregularity of periods... Having these is not good for our health, that is my opinion, it affects very badly.”* (Respondent CJ).

*“This was initially not a problem for me but recently, my weight gain affected me a lot.....emotionally. And then when I get a bit bothered about my appearance, I feel bad. Sometimes.... I think why me, but then usually I find some ways to comfort myself.”* (Respondent AL).

Although in their study, Sulaiman et al. (2017) indicated that PCOS syndrome was associated with a higher risk of psychological burden, future research, in consultation with the present study findings, could address these psychological needs of the PCOS population to manage the syndrome effectively. Consequently, the current findings address the unmarried women’s perception of the syndrome, which considerably reflects their psychological needs in their effective dealings with PCOS. They indicate the benefit of sharing a common burden in working to better deal with the conditions of the syndrome with peer support. The present findings partially support the past observation of Amiri et al. (2014) that women with PCOS tend to have an inferior feeling in comparison to women with no PCOS, as their feminine feeling is reduced through the syndrome, and that the PCOS affected women are further disturbed psychologically and emotionally. However, going beyond the disturbances and psychological distress as perceived in a larger cultural context such as South Asia (Kumarapeli et al., 2011), the present study calls for an integrated approach to offer the much needed psychological and emotional support to the reproductive age women in their early adulthood, especially in the context of multicultural setups such as India.

#### 3.2.2 Sub-theme two: perception towards PCOS treatment

Participants shared that they have known women with PCOS to avoid medications after the diagnosis. In their understanding, despite possible benefits of the allopathy medicines, the avoidance resulted from perceived non-progress of health conditions, reduced belief in the medicinal effects, and perceived side effects and higher costs of medicines. Further, they perceived that they feel frustrated with regular consumption and that they do not like to take medicines anymore. In addition, the unmarried women expressed that they perceived consulting a doctor for PCOS treatment as disinteresting. It mainly resulted from avoiding the scolding for having not made necessary changes in their lifestyles.

*“In my circle I have known women with PCOS not consistently taking medicines; in between, they will stop... I perceived that they feel there is no reduction in the disease, in such cases even I would quit that medicine… like I don’t believe in these medicines…. I thought that there were no uses in taking the medicines. Allopathic medicines are for no good and in turn cause side effects. And the cost of these medicines is also high..”* (Respondent BP).

*“It is too frustrating and irritating. I really don’t like to have tablets..... I am like when will I get out of this....when will I get rid of this. I really don’t like this.”* (Respondent LJ).

*“I am not so interested in consulting a doctor because she would scold me if the necessary changes in lifestyle were not made.”* (Respondent CG).

These findings have further added up to the list of possible reasons for such avoidance (Taber et al., 2014); unfavourable evaluation of medical care, primarily resulting from concerns over side effects, physicians and healthcare-related factors; b) lesser perceived need over anticipation of recovery over the period, c) traditional barriers such as time constraints and higher medication costs. In contrast, few participants who were prescribed medicines claimed that the doctors instead advised them to follow a healthy lifestyle to avoid dependency on the medications. The findings go beyond highlighting that women with PCOS are considerably aware of the how abouts of the syndromes to report the difficulties faced in indulging regular consumption of medications. The perceived problems as shared by the study participants are of great relevance for further exploration that aims to promote effective multidimensional management of the syndrome.

### 3.3 Theme three: changes experienced due to PCOS

This theme includes the physical and cognitive changes as its sub-themes. The study respondents have shared that they mainly have experienced physical and cognitive changes as part of their PCOS condition. The major physical changes observed and reported were weight gain, acne on the face, hair loss, developing ulcers etc. While the major cognitive changes observed were disturbed attention and concentration. The relevance of these changes, especially from the perspective of unmarried women, was discussed in the respective sub-themes as follows:

#### 3.3.1 Sub-theme one: physical changes caused by PCOS

Among the physical changes, the participants' most common change due to PCOS was weight gain. Some are said to have gained it due to medicines. In comparison, few others were confused about their reason for weight gain. Besides, some visible changes were pimples and acne in the face, unwanted hair growth or hair loss, dark patches on the skin folds, and unstable mood swings, which were very disturbing for some patients.

*“While taking allopathic medicines, I gained so much weight and developed an ulcer”* (Respondent BP).

*“Body.... I said that I was underweight and due to PCOD I gained nearly 10 kgs. I guess that it is due to the medication that I am taking. And I have noticed hair fall also and also overgrowth of hairs in some parts of the body.”* (Respondent LJ).

*“Of course I had hair growth in my inner parts and also hair fall in head and irregular periods and over flow and slight mood swings in the initial stage.”* (Respondent CJ).

*“Yes, I guess there were like loss or thinning of hair excess of unwanted hair dark pigmentation on neck and nether region and also periods that decides to come at the right times and even way past the due date, at different intensities and quantity.”* (Respondent PMP).

As observed in the past, the current study findings also highlighted the body-associated changes mainly were the alterations in weight, especially weight gain, eventually resulting in obesity, a more significant risk factor for several other associated illnesses such as cardiovascular syndromes and diabetes mellitus etc (Thomson et al., 2011). Moreover, although past studies such as of El Hayek et al. (2016) provided an updated overview of the syndrome, especially the broader categories of associated morbidities such as obesity, insulin resistance, type II diabetes, cardiovascular diseases, infertility and cancer, especially from adolescents perspective, the current study offered insights onto the specific physical changes caused by PCOS among unmarried women of southern parts on India. Given that these physical changes could induce psychological issues that may affect an individual's self-esteem and other aspects, the current study emphasizes adequate clinical attention to effectively address and deal with the physiological changes. Interestingly, the unmarried women in their early reproductive age and who are most likely to get married in the near future haven't shared the concern of infertility concerning their present physical condition. This finding paves the way for further exploration in this regard.

#### 3.3.2 Sub-theme two: cognitive changes caused by PCOS

Participants’ experiences of cognitive changes caused by the PCOS indicated the disturbances to mainly the cognitive abilities, such as attention and concentration, while for the changes in thinking abilities and decision-making, the study respondents have had differing opinions. Some have agreed to have experienced the changes in that two aspects, while a few have not experienced the same. At the same time, a few more respondents who have experienced such changes were not sure if those changes were resultant of PCOS condition.

*“Although I feel my decision-making is all perfect. But I think sometimes I lose my concentration and attention a little. I can’t listen to all things what teacher says in class. Before that and all, I felt that I was more concentrating. I don’t know whether this is the reason or not.” (LJ). “I don’t know whether this is because of PCOD or not, but I think that my academic performance has reduced and my attention and concentration has reduced. I didn’t notice any changes in thinking and decision making..”* (Respondent AL).

*“Yes, I am so confused now a days and I am having difficulty in making decisions. If someone is talking more, I could not keep my attention there and I feel like I am not concentrating on anything for so long like usually how I was before. ”*(Respondent SR).

However, past studies on assessing the influence of PCOS on the cognitive abilities of the syndrome found no substantial conclusions for the same (Jarrett et al., 2019; Lujan and Mergler, 2015). Although Lujan and Mergler (2015) found reduced levels of spatial ability among PCOS patients, they warranted further studies to ascertain meaningfully investigate the impact of cognitive task performance among PCOS patients. With the prevailing ambiguity, the current study sheds light on further research, especially empirically exploring the same effect via a series of experiments.

### 3.4 Theme four: perceived side effects of PCOS medications

The medicines, and predominantly the side effects of allopathic medications, were of significant concern among the PCOS patients in our study findings, especially in this major theme. At the same time, some opined that these medicines were beneficial at times apart from the side effects. However, the respondents reported the primary concern of consumption was related to side effects such as weight gain, headaches, mood swings, ulcers, etc.

*“I am not taking any medicines for PCOS, because the medication for PCOS is mostly hormones, and I don’t want any kind of hormonal imbalances in my body and cause side effects.”* (Respondent KT).

*“I also think that non-hormonal tablets also may have some side effects on my body and while taking ayurveda, it was okay for four months, everything was regular and then I thought of stopping it. At present, it gets a bit delayed.”* (Respondent BP).

*“Periods were regular when I took the tablets but once I stopped, it was again irregular. Then gradually I thought that hormonal tablets would give me side effects and all, so we decided to switch to homeopathic medicines. It is also same, would be regular if we consume it regularly and if not, she would again give medicines for that.”* (Respondent AL).

Similarly, from the past studies, diverse observations and experiences among PCOS patients regarding relevant medicines' benefits and their adverse effects. In their meta-analysis, Domecq et al. (2013) pointed out that no relevant studies reported significant severe side effects, including weight gain resultant from commonly used medications for PCOS syndrome. Bashir et al. (2021) found that although Allopathic, Unani and Ayurvedic medicines showed good outcomes in management and treatment of the syndrome, most patients, due to tremendous side effects medications. Instead, they often opt for Unani or Ayurvedic medicines. The current findings also emphasized unmarried women's personal views, which seemed to have overtaken these observations and preferences of type of medication. Their primary concern was about hormonal imbalance; hence, their choices were determined accordingly.

### 3.5 Theme five: perceived role of social factors for effective management of PCOS

The fifth major theme emphasized the perceived role of social factors such as society’s attitude and the role of perceived social support in the effective management of PCOS among unmarried women of early reproductive age group from the multicultural perspective, specifically in the southern part of India. The results of two sub-themes have been presented and discussed as follows:

#### 3.5.1 Sub-theme one: role of society’s attitudes

The significant *role of society’s attitude* as a sub-theme of the main theme –*perceived role of social factors for effective management of PCOS* includes the perception of the individuals upon the thoughts of their community over PCOS syndrome. The respondents seem to have taken a serious note of the syndrome resulting from their pinch of closeness towards maturity led by forthcoming social and family pressures or responsibilities and expectations such as getting married soon.

*“When I was diagnosed with PCOS, the first thing that my mum told me is that please don’t tell this to anyone. I am the kind of person who tells anything and everything to all other people, and she wanted me to shut my mouth when this topic comes. So, I was like, why did you say that, then she said that if someone comes to know that you have PCOS, then in future, when marriage proposal and all comes, then this might cause problems.”* (Respondent LJ).

*“Actually my parents have told me to keep quiet about it. I guess culture is playing a big role, in this forward thinking society…. This old generation people they are having an inhibition in taking medication for PCOD or saying others about having PCOD… such things I guess play their role”* (Respondent LP).

*“I think that they believe that women are essential for the future generation. It may lead to infertility, so women are more anxious about our future. Maybe we may become infertile. And they are like pressurizing to go and take medicines and sympathizing”*. (Respondent BP).

Individuals, especially in Indian society, seem to have a set of misconceptions and stigma associated with this issue. Sadly, many people don’t even attempt to talk freely about PCOS. Revealing PCOS diagnosis is superstitiously believed to cause a problem for future marriage. Predominantly, such attitudes seem to stem from a misconception of the syndrome. One of the significant reasons would be related to the fertility of the female. Provided the Indian cultural aspects, the carry-forward of the respective bloodline for the coming generations, considerable family members tend to be quite worried of such complications and, therefore, more likely to hide such critical information. On the other hand, several PCOS patients were greatly concerned about their treatment for the syndrome because women are predominantly meant to be essential means for future generations, i.e., the offspring.

This sub-theme partially supports the findings of Sharma and Mishra (2017) who explored the PCOS as a ‘tabooed disease’ in light of cultural explanation – the disease is resultant of black magic and is a source of inviting shame onto the family. Especially about the concern of fertility among parents and close ones of the unmarried women aptly describes the cultural aspect highlighted by Sharma and Mishra (2017) that mothers-in-law perceive this condition of a woman (a potential daughter-in-law) as being totally incapable of bearing a child. The current study, especially from the perspective of unmarried women, highlights their parents' concerns regarding the infertility issues that could lead to missing out on potential matches for the marriage. Thus, the present research indicates a culturally relevant, multidisciplinary method to offer health education, counselling, and comprehensive care for unmarried women with PCOS and their respective support systems, such as family and friends.

#### 3.5.2 Sub-theme two: role of perceived social support

In contrast, *role of social* support as another sub-theme emphasized that that an individual's support from society is necessary for effectively managing the PCOS. In general, as the participants reported, the study observed a lack of awareness of syndromes such as PCOS among people in the community.

However, on the other hand, PCOS patients also reported that post-diagnosis, their family members were very supportive of them in every aspect. These supporting roles of family members ranged from helping to maintain a healthy diet and regular exercise. Besides, their friends were also of more incredible help. They supported, considering their role in taking them to hospital for a consultation, advising and helping in maintaining healthy eating habits and engagement in regular physical exercise. However, a few respondents shared that their support was mainly driven by improving their physical difficulties and well-being, while the family members did not well support the psychological distress caused by the syndrome. Whereas in a few other cases, although parents supported unmarried women, they couldn't continue the support due to their non-adherence to structured exercises and diet practices.

*“Great!! They are always a big support for me in everything. They help me to keep up my diet. In fact, they also have reduced eating and buying junk food items so as to encourage me to follow good food habits. And I also really like to eat non-vegetarian and I used to eat it without any limitations but now I have started controlling my food habits.”* (Respondent CG).

*“Both my friends and family are quite supportive and are not embarrassed. I think that PCOD causes problems later in pregnancy and all… that’s why family members are so much bothered about it… I think that most other parents are concerned more about only the physical problems caused by PCOD, and don’t understand much about the mental distress that it causes.”* (Respondent AL).

*“They pressurised me to go to doctor and take medicines. They help me in taking those medicines and then in maintaining diet. They then tell that it is your body, you have to take care of it. They are supporting me but because I stop medicines in between and don’t care much about exercise and diet, they left it all to me. They are fed up with me.”* (Respondent BP).

The current study findings for perceived social factors are significantly interesting in highlighting that the social support received, especially by unmarried women's family and friends, relates to past studies such as Navid et al. (2018). They had observed no significant differences between infertile women with and without PCOS concerning to their marital satisfaction and social support. Although the current study findings' comparison of married and unmarried or fertile and infertile women is beyond the scope, it could be argued as follows based on the perceived social support towards PCOS condition. Infertility is more considerably problematic than PCOS per se, and the more severe condition of PCOS could lead to infertility. The social stakeholders seem to address the issue and offer all possible support as early as pre-marital life.

### 3.6 Theme six: past experiences of PCOS medications

This central theme includes the individual’s experiences with the treatment, changes after taking medicines, and unique experiences with e the PCOS medications. In the opinion of PCOS patients, the medicines consumed by them were pretty notably helping them effectively manage the conditions. Apart from side effects in allopathy and slower effects in Ayurveda, they were mainly inclined towards medicine consumption as prescribed by the doctors.

*“Yeah, there was a huge change… now it is like 28 plus or minus two. It has become very predictable, but earlier, before diagnosis, this was difficult.”* (Respondent APM).

*“Yes, it was regular. It was also regular after taking the hormonal tablets, but weight gain was the problem and ulcer. And I also have migraine already, and when I take those hormonal tablets no....headaches comes .... hormonal ones, not non-hormonal and Ayurvedic. Ayurvedic is better.”* (Respondent BP).

*“Yes, very much important, I think. as I don’t exercise regularly, because of tablets only I am getting proper periods.”*(Respondent ABC).

*“I have not taken any medications for this from the starting. I think that taking those medicines will only give hormonal imbalances and other side effects. I think that there is no need to take medicines because it is manageable for me.”* (Respondent KT).

As reported, significant reasons for avoiding medications are the aftermath of consuming such medicines subject to hormonal imbalance. This finding was aligned with previous findings (Taber et al., 2014), which reported that a lower perceived need to take medicines and unfavourable evaluations of drugs could result from the very medical avoidance among PCOS patients. Upon regular consumption of prescribed medicines, the patients shared that their menstrual cycles eventually became more predictable and stable, the most frequent and common change reported among the study participants. It also helped resolve several common issues such as headaches, pimples, mood swings and Premenstrual Syndrome issues, stomach pain, heavy bleeding etc. On the contrary, a few PCOS participants opined that consuming medicines caused undesirable effects such as increased headache (previously had a migraine), weight gain, ulcers etc. Interestingly, although the current findings of desirable effects of medicines were also defiant to such results in the past (Domecq et al., 2013) specific segment of the study participants also reported the severe side effects of medications over the period, especially for the patients who had associated issues such as headache and migraine.

## 4 Conclusion

Many of the participants in the study expressed similar perceptions toward the importance of diet, exercise and a healthy lifestyle in maintaining PCOS and overall health. In general, a lack of awareness in individuals diagnosed with PCOS at a younger age was seen; however, upon increased age and associated maturity, they seemed to have taken the necessary measures to alter the situation considering the forthcoming social and family responsibilities including marriage and conceiving a child. Further, awareness among individuals in society was also found to be insufficient. These observations highlight the cultural relevance of perceived social pressure on the family of unmarried women with PCOS.

Essentially, medicines helped get a regular menstrual cycle but had side effects on the health, as reported by a few participants. Besides, it was also observed that all the participants had reported some unhealthy behaviours in their lifestyle even before the diagnosis. Most of them were consuming an unhealthy diet, lacking regular physical exercise, and now were attempting to change their lifestyle post-diagnosis.

## 5 Implications

Unmarried women with PCOS in comparison to married women with PCOS are at higher risk of being obese and overweight and are generally more likely to be anxious and depressive during their reproductive ages. Thus, the current study, especially for unmarried women in their reproductive age, offers valuable insights into dealing with the complexities of hormonal imbalances, reproductive health, and anticipation of marriage and fertility shortly. In doing so, primarily their knowledge health care behaviours and necessary lifestyle changes apart from social and family support, play a crucial role.

The qualitative inquiry offers relevant insights into further investigation of appropriate integrative interventions to deal with the complexities of the symptom and psychological issues, primarily about near-future adult responsibilities. Being the first of its kind, the study's qualitative approach sheds light on the cultural context of the non-clinical management of PCOS, especially among unmarried women of reproductive age groups.

## 6 Limitations and suggestions for further research

Predominantly, due to the pandemic related restrictions and low participation rate, the present study was limited to a small sample size. The interviews for the intended data collection were confined to fifteen respondents on the pragmatic grounds rather than saturation determination. Hence, it could be noted that further interviews may have offered additional themes. In addition, characteristically, the qualitative nature of the study limits its generalization. Due to the lack of data especially from the married women with PCOS, the comparison of perception, knowledge and attitudes between married and unmarried women could not be made. However, the present study warrants future studies to emphasize: a) adapting to a multidisciplinary and mixed methods approach to investigate the unanswered questions from the study, b) exclusively exploring the cultural attributes of unmarried and married women’s perception and attitudes towards their PCOS condition and effective management.

## Declarations

### Author contribution statement

Eslavath Rajkumar, PhD: Conceived and designed the experiments; Performed the experiments; Analyzed and interpreted the data; Wrote the paper.

Ardra A, B.Sc: Conceived and designed the experiments; Performed the experiments; Analyzed and interpreted the data; Wrote the paper.

Prabhu G, M.Sc: Conceived and designed the experiments; Analyzed and interpreted the data; Wrote the paper.

Dr. Vijyendra Pandey, PhD; Dr. Jeyavel Sudaramoorthy, PhD: Performed the experiments; Analyzed and interpreted the data.

Dr. Rameez Manzoor, PhD; Dr. Sooraj K V, PhD; Dr. Manikandaprabu M, PhD; Tukaram Badiger, MSW: Contributed reagents, materials, analysis tools or data.

### Funding statement

This research did not receive any specific grant from funding agencies in the public, commercial, or not-for-profit sectors.

### Data availability statement

Data will be made available on request.

### Declaration of interests statement

The authors declare no conflict of interest.

### Additional information

No additional information is available for this paper.
